# Global Strategies for Population Management of Domestic Cats (*Felis catus*): A Systematic Review to Inform Best Practice Management for Remote Indigenous Communities in Australia

**DOI:** 10.3390/ani10040663

**Published:** 2020-04-11

**Authors:** Brooke P. A. Kennedy, Bonny Cumming, Wendy Y. Brown

**Affiliations:** 1 AMRRIC—Animal Management in Rural and Remote Indigenous Communities, Winnellie, NT 0820, Australia; Bonny.cumming@amrric.org; 2 School of Environmental and Rural Science, University of New England, Armidale NSW 2353, Australia; wbrown@une.edu.au

**Keywords:** domestic cat, *Felis catus*, population management, indigenous community, aboriginal community, culturally appropriate

## Abstract

**Simple Summary:**

A systematic review process was used to identify the current global cat population management practices for owned, free-roaming cat populations, particularly those suited to remote Indigenous communities in Australia. Based on this review, a combination of three methods is recommended. The first method, Surgical Sterilisation (SS), requires owners to take their socialised cats to a facility for surgical sterilisation and then take them home. The second method, Trap-Neuter-Release (TNR) traps, neuters (sterilises) and returns healthy owned cats, which are unsocialised to the point where trapping is necessary, to their homes, and the third method, Trap-Remove (TR), traps and removes unwanted social cats via adoption and unhealthy cats via euthanasia. Conducting these three methods together over a long-term period appears to be consistent with current global best practice for humane and ethical management of cat populations in these communities.

**Abstract:**

Pet domestic cat (*Felis catus*) populations are increasing all around the world, resulting in an increase in contact with humans and wildlife, potentially spreading zoonotic diseases and predating on wildlife. With the recently identified rise in cat populations in remote Indigenous communities in Australia, culturally appropriate cat population management strategies are required. A systematic review process was conducted to review the current global cat population management practices that are suitable for owned, free-roaming cat populations in these communities. Eight articles on in-situ field cat populations and five studies simulating computer modelled cat populations reported results of 66 population management interventions. Surgical Sterilisation (SS) was used in all socialised owned cat articles. The trap–neuter–release (TNR) method was used most frequently on unsocialised cats and gained the best results when the trap–remove (TR) method was used concurrently to adopt out unwanted social cats and euthanise ill or injured cats. The results of this review suggest that long-term TNR/SS programs supplemented with TR provide the current most ethically sound best practice, humane method of managing cat populations in remote Australian Indigenous communities. It is also recognised that no one plan will fit all, and that further research on the micro-level techniques used to deploy both TNR and TR needs to occur, and that culturally appropriate community consultation during all processes is vital in achieving a sustainable management program.

## 1. Introduction

The domestic cat (*Felis catus*) can be found on every continent except Antarctica, including many islands, with feral cats spreading far beyond their areas of introduction [1] (p. 51). The global cat population was estimated at around 600 million in 2009 by Dauphiné and Cooper [2]. However, with pet cat numbers increasing all around the world, this is now widely regarded as a gross underestimate [1] (pp. 51–52). This increase is most likely due to the high fecundity of cats, particularly those whose food is supplemented by humans [3]. On average, female cats produce four kittens twice a year, (ranging from 1–10 kittens and 1–3 litters per year) resulting in 40 kittens over a 5-year lifespan [1,4,5,6]. Increasing populations of cats that are in contact with humans also increases human health risks, with many cat-associated zoonoses ranging from trivial to fatal (see review by Kravetz and Federman [7]). Additionally, free-roaming cats (owned, semi-owned or feral) can have negative impacts on biodiversity: these include predation; competition hybridisation and transmission of disease (see review by Woinarski, Legge and Dickman [1]; pp. 51–69). 

In Australia, feral cats occur across more than 99.8% of Australia’s land area (including islands) and their populations fluctuate in natural environments with seasonal rainfall from 1.39 million in dry/drought conditions to 5.56 million after extensive rain, with an average of about 2.07 million across good and bad seasons [8]. The pet cat population in Australia is currently estimated at 3.76 million, occurring in 27% of Australian households [9]. There are also an estimated 1.2–2 million stray (or semi-owned) cats in urban Australia [10]. This equates to an estimated total 7.03–7.83 million cats in Australia, ranging from 6.35 million in dry/drought conditions to 11.32 million after extensive rain. 

In remote Indigenous Australian communities, pet cat populations are increasing [11,12], adding to the total cat population of Australia. However, the style of ownership in these communities is quite unique. Pets (including cats) can be owned by an individual, by a family or by a family group and therefore can have multiple households considered as ‘home’ and are usually allowed to roam free. In Western society, this may be seen as a lack of owner responsibility. However, in these communities, companion animals are treated as children rather than pets (not the same way Westerners see their “fur babies”); the pets need to learn to be responsible and must accept any consequences of their actions. The relationship is one of companionship, in the true sense of the word, rather than ownership. 

Although cats can be valued pets, they can also pose significant risks. Within Australia, remote Indigenous communities are often located in largely natural environments with high biodiversity values. With the majority of cats in these communities being free-roaming, they access these areas to hunt native wildlife, readily able to become feral, breed and spread disease to and predate on wildlife. A high prevalence of feline-borne parasites, including hookworm (*Ancylostoma tubaeforme*) and toxoplasmosis (*Toxoplasma gondii*), have been identified in remote Indigenous communities in Western Australia, posing a zoonotic risk, especially for children living in these communities [13]. 

It is generally accepted that there is a need for cat management, but that it must be humane [14]. Whilst much of Australia is lacking in coordinated cat management strategies, it has been suggested that feral cat management activity is greater in Australia than any other continent [1] (p. 169). There have been multiple reviews on feral cat population management in Australia [1,15,16,17]. The most recent review provides details on a range of current cat population control methods; cat exclusion (fencing and islands), poison baiting, trapping, shooting and hunting, habitat management (i.e., fire management), managing prey (i.e., reducing/removing rabbit and/or rodent populations to reduce resources for cat populations), managing predators (i.e., meso-predator control), trap–neuter–return (TNR; sometimes termed trap–neuter–release), managing pet cats and urban feral cats and biological control (immunocontraception, disease). Emerging management strategies are also mentioned, such as guardian dogs and new poison delivering systems as well as next generation possibilities such as controlled selection for predator awareness and biological control through gene drives ([1] Chapter 9). However, many of these management strategies are not suitable for managing cats that have a relationship with a human owner or caregiver. Certainly, those that involve eradication through mass culling or removal are likely to cause significant trauma and distress to owners. Furthermore, when it comes to remote Indigenous communities, there is the possibility of a strong cultural/spiritual connection that also needs to be taken into account in any cat management practice. For example, in remote Indigenous Australian communities, some pets are named after family members that have recently passed away, and to euthanise a “named” animal, would be equivalent to euthanising the family member they have lost, which could cause serious implications [18].

Animal Management in Rural and Remote Indigenous Communities (AMRRIC) is a national, not-for-profit organisation that uses a One Health approach to coordinate veterinary and educational services in remote Indigenous communities. With sparse populations and limited opportunities to successfully operate small businesses locally, most remote Indigenous communities have limited access to veterinary services. Where available, vet services are usually delivered on a quarterly to biannual basis, by visiting vet service providers. These services are typically funded by the local government authority and provided free of charge to community members. In response to identified need, much of AMRRIC’s work historically has focused on providing guidance and delivering effective and humane dog population management programs. With the recently identified rise in cat populations in these communities, culturally appropriate cat population management strategies are required. The first step in developing such strategies is to review the current global cat population management practices that would suit the owned, free-roaming cat populations AMRRIC are engaged with, that meet the ethos and cultural practices of the cats’ Indigenous owners. This review aims to address this need.

## 2. Methods

### 2.1. Search Strategy

A systematic review was conducted using the Google Scholar web search engine (31 October–13 November 2019). Google Scholar was selected as it not only includes most peer-review articles, but it also includes conference papers and abstracts, technical reports, theses and dissertations, academic books and pre-prints from major and lesser known sources from all around the world [19]. The following search terms were used: “cat” OR “feline” AND “population management” OR “population control” AND “owned” OR “semi-owned” OR “community” OR “free roaming.” To determine current practices, the filter “since 2015” was also used. 

### 2.2. Selcetion of Articles for Review 

A database search of Google Scholar returned a total of 987 articles. Each article was independently critiqued by Kennedy in a step-by-step process using exclusion criteria for eligibility (Table 1). Articles were included if a method of intervention was implemented to control an in-situ population of domestic cats, whether owned, semi-owned, stray or feral, as defined by RSPCA Australia [20]. Criterion 5 refers to the population control method being implemented. Articles that described population control methods that are not yet registered and approved for use in cats were excluded: for example, the testing of new long-term contraceptive drugs. Criterion 6 also relates to the method being used. The residents in the communities that are serviced by AMRRIC do not accept mass euthanasia/culling as an appropriate population control tool, as it would involve removing their pets without permission [21], causing trauma and distress, with greater implications if “named” animals, as described above, were removed. Therefore, articles that implemented only population control methods involving mass culling/euthanasia, were also excluded. Articles were excluded if the following were not reported in the methods (Criterion 7): population control method, duration of implementation, frequency of intervention, description of cat type and the environment in which the population resides. 

## 3. Results

**Figure 1:**
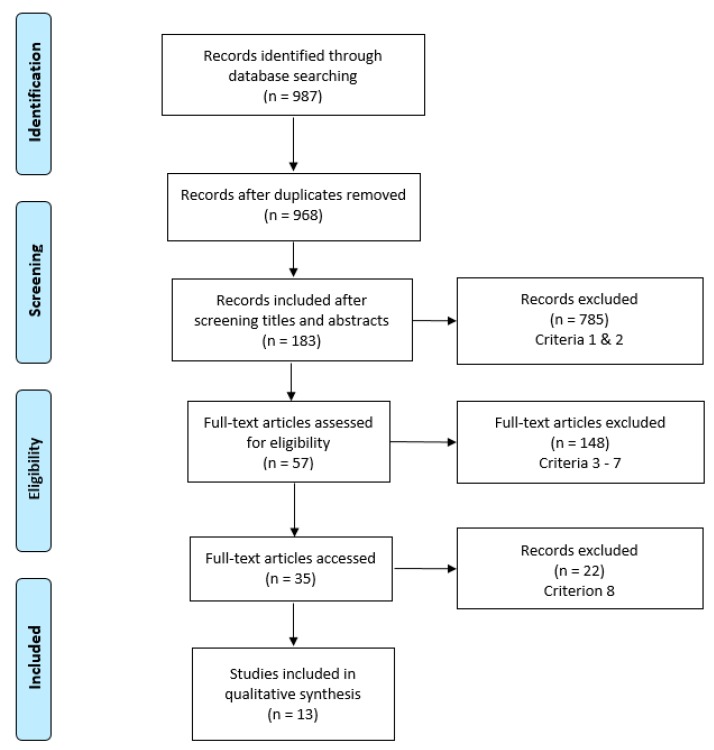
Number of papers included and excluded throughout the stages of the systematic review process. Descriptions of each criteria are reported in Table 1.

A total of 13 articles were eligible for inclusion (Figure 1). The relevant articles were divided into two separate categories—field work and simulations—and are reviewed separately below. The field work articles were those where the management strategy was implemented in field environments, where the cats normally reside. Simulation articles utilised computer models to predict population changes. Some simulation articles incorporated field work to estimate existing populations and demographics such as gender ratios, percentages of sterilized animals, and population turnover rates as inputs for their models; however, the execution of the management strategy was completed using computer models. 

### 3.1. Fieldwork

Eight of the thirteen articles evaluated cat population control programs that were conducted in the field, and these are summarized in Table 2 and detailed below. These articles looked at owned and free-roaming cats in urban and rural environments in 5 different countries. A variation of TNR—a method where an animal (in this case, a cat) is trapped, taken to a facility for surgical sterilisation and then released at the location of the original trapping [22]—was the method of population management for all free-roaming cat population articles (n = 4). Surgical sterilisation (SS)—a method where cat owners transported their pet cat(s) to a facility for surgical sterilisation and then transported them back home [23]—was used in the owned cat population article (n = 1). 

#### 3.1.1. Surgical Sterilisation (SS)

Robbins, et al. [23] evaluated the effectiveness of a subsidised SS strategy on an owned cat population in an urban environment. As a result of this strategy, there was a significant (*p* = 0.01) increase in the number and proportion of kittens neutered from 17 (20% of kittens < 6 months old) to 47 (39% of kittens < 6 months old).

#### 3.1.2. Short-Term TNR Programs

Three studies evaluated TNR programs that ran for 12 months or less in New Zealand, the US and Canada respectively [14,22,24]. The Auckland study [14] evaluated their program by assessing the intake of stray cats into shelters in each of two designated suburbs (targeted and control) before and after the 12-month TNR program occurred in the targeted suburb. There were significant differences (*p* < 0.01) between the control and targeted groups’ intake of adult cats and juvenile cats (control: increased by 17% adults and 43% juveniles; targeted: decreased by 39% adults and 17% juveniles). There was also a significant difference (*p* < 0.01) between the control and targeted groups’ euthanasia of adult and juvenile cats (control: increased by 13% adults and 43% juvenile; targeted: decreased by 47% adults and 34% juveniles). In the New York study [22], free-roaming cat populations were estimated before and after an 8-month TNR program. No significant difference was found in the total population estimates (t = −0.8238, df = 3, *p*-value = 0.47), despite a significantly greater proportion of ear-tipped (sterilised) cats in the treatment sites compared to the control sites (control*treatment: χ^2^ = 5.7701, df = 1 *p* = 0.016). A significant difference was found between the treatments and the year (year*treatment interaction: χ^2^ = 5.7715, df = 1, *p* = 0.016), suggesting that the number of neutered cats increased significantly in treatment sites after the TNR program. The Canadian study [24] trialled a one-off TNR event across 10 colonies and reported cat populations at T_0_, T_7_ (7.5 months after event) and T_12_ (12 months after event) compared to eight control colonies. At T_0_, a median of 95% (range: 67% to 100%, mean: 92%) of the cats in the TNR group where trapped, neutered and returned. At T_7_, the (adults only) control group had increased by a median of 2.5 adult cats per control colony which was significantly greater (*p* = 0.03) than the TNR group where no change in population was observed. However, at T_12_, no significant differences of the number of adults were observed between the TNR and control colony populations. The number of kittens only and the numbers of kittens and adults combined, showed no differences at T_0_, T_7_ or T_12_.

#### 3.1.3. Long-Term TNR Programs

Four articles assessed the impact of long-term conventional TNR programs on free-roaming cat populations in the United States [25,26,27], and in Australia [28], over extended periods of time; 23, 15, 10 and 9 years respectively. A US study [25] retrospectively evaluated a 23-year TNR program through census data collected before and during the program (program is ongoing). The population decreased significantly from 455 in 1999 to 206 in 2013 (decrease of 55%, *p* < 0.0001), with, over the period reported, a total of 3487 cats involved: 1111 returned, 510 adopted, 210 transferred to adoption centre, 441 unhealthy/retrovirus-positive cats euthanised, 58 dying in care and 209 dead on arrival. The Newburyport study [26] trapped, neutered and either returned, adopted or euthanised more than 300 cats with the majority being part of the initial population (~300) and ~40 additional cats that migrated to the population during the management program. With 100% of the cats at Newburyport neutered during the program, the last cat passed away of old age (~16 years old) 15 years after the beginning of the program, resulting in a decrease in 100% of the cat population. In a citizen science study in Illinois, USA [27], a TNR program was conducted across 20 colonies in a 9.3km^2^ urban area. A total of 75 baseline cats and 120 migrant cats (or kittens born) were trapped, neutered and either returned (44), adopted (59), disappeared (67), died (6 euthanised, 13 other causes), returned to owner (3) or were relocated outside the area (3). Eight colonies decreased by 100%, four colonies showed 0% change, five increased by > 50% and three decreased by <50%. This high variation in colonies is most likely due to the variation in population size of each colony; however, overall, there was a population decrease of 54%. In the Australian University Campus study [28], a similar method was undertaken, with 69 baseline cats and 34 immigrants all trapped, neutered and either returned (85) or rehomed/returned to owner (18) during the program. By 2009, only 15 neutered cats remained, resulting in a decrease of 78% of the cat population. 

**Table 2:** Results from eight articles where a cat population management strategy was implemented in a field environment.

Ref.	Location	Date	Duration	Frequency	Description	Environment	Method	Results	*p*-Value
[14]	Auckland, NZ	2015–2016	12 months	Throughout 12-month period	Free-roaming	Urban	TNR	Intake of adult strays: control up 17%, TNR down 39%	*p* < 0.01
								Intake of Juvenile strays: control up 43%, TNR down 17%	*p* < 0.01
								Unsocial strays neutered and returned: control up 100%, TNR down 7%	*p* < 0.01
[23]	UK	2013-2014	12 months: 6 months before and after	Continual for 6 months	Owned	Urban	SS	Proportion of sterilised kittens increased from 20% to 39%	*p* < 0.01
[22]	New York City, US	2011–2012	8 months	Continual for 8-month	Free-roaming	Urban	TNR	No significant effect on population count	*p* = 0.47
[24]	Quebec, Canada	2014–2015	12 months	One-off at T_0_	Free-roaming	Rural	HI TNR	Significantly fewer adult cats in TNR colonies after 7 months. No difference at 12 months.	*p* = 0.03
[26]	Newburyport waterfront, Mass, US	1995–2009	15 years	Continual	Free-roaming	Urban	TNR/R	Population decreased by 100% after 15 years	
[28]	UNSW, Australia	2008–2017	9 years	Continual	Free-roaming	Campus	TNR/R	Population decreased by 78%	
[25]	Ocean Reef, Florida, USA	1995–2017	20 years	Continual	Free-roaming	rural	TNR/R	Population decreased by 55%	*p* < 0.0001
[27]	Chicago, Illinois, US	2007–2016	10 years	Continual	Free-roaming	Urban	TNR/R–20 colonies	Average 54% decrease in populations	

### 3.2. Simulations

Five articles used simulation models to predict cat population changes, using multiple methods at differing intensities (Table 3). Two studied an owned cat population, two concentrated on free-roaming urban cats, and one was focused on an island population of cats including both owned and free-roaming strays. 

#### 3.2.1. Simulations Using Field Work Data as Input Models

Lancaster, et al. [29] used county-specific literature to create population model data for a cat population within their county. Using this data, the authors simulated the effects of increasing the intensity of the SS program (50%, 75% and 100% more sterilisations) focusing on different age groups in the population. They found that undertaking SS with 50% more queens between the ages of 4 months and 5 years of age on top of the current spay rate to be the most cost-effective method, increasing the number of sterilisations by 2.8% from 36,345 spays to 37,359 spays in a population of 55,000 female cats. Similarly, Dias, et al. [30] used data from a local sterilisation campaign to create their simulation model. A household survey was conducted before and after a campaign where free SS was offered, to ascertain the proportion of sterilised cats, revealing a 0.68% increase from 21.89% to 22.57% sterilised after campaign. This data was modelled, and a range of simulations were conducted, with the authors concluding that running the current campaign annually was the most cost-effective solution, resulting in a predicted sterilised rate of 75% after 20 years. 

#### 3.2.2. Simulations Using Literature and Expert Opinion as Model Inputs 

Three articles used literature and expert opinions as data inputs for their simulations and compared multiple methods of managing free-roaming cat populations. In an urban population of 50 cats, Boone, et al. [31] simulated low (25%) and high (50%) intensities for; no management (control), trap–cull (TC, euthanasia), trap–neuter–return (TNR), and trap–remove (TR; euthanasia or adoption). TC results are not presented in Table 2 as they do not meet Criterion 6; however, they are presented here for comparison. All simulated management methods resulted in a decrease in population size at 25% intensity (Control = 0.94%, TC = 10.6%, TNR = 22.5% and TR 47.26% reduction) and at 50% intensity (Control = 0.94%, TC = 21.98%, TNR = 48.4% and TR = 86.4% reduction). In this article, the carrying capacity of the population was 50 individuals; during the TC program, a cull took place whenever this population was reached. This also explains why the control program did not result in a population increase. Miller, et al. [32] simulated four different management programs; TR, TNR, contraceptive A (ConA; temporarily sterilised for 3 years) and contraceptive B (ConB; temporarily sterilised for 6 months to 5 years). ConA represents a hypothetical program, as currently there is no contraception available that is guaranteed to last up to three years. ConB mimics laboratory results of GnRH immunocontraception, GonaCon injections, which has been reported to last between 6 months and 5 years. Neither ConA nor ConB have had their safety or efficacy evaluated as a part of a population management method, nor have they been registered or approved for use in cats and therefore were not presented in this review (see Criterion 5). This article simulated these four management methods in demographically isolated and demographically connected (abandonment and dispersal) large urban, small urban and rural free-roaming cat populations. Only those that are relevant, currently plausible (given existing contraceptive methods) and evaluated are presented in Table 2. In isolated rural populations, a minimum of 20% removed (TR) every 6 months and 10% surgically sterilised (TNR) every 6 months resulted in population elimination. In both small and large urban areas, TR minimum 30% every 6 months and TNR minimum 40% every 6 months both resulted in a consistent decline in the population, with 40% TNR resulting in a long-term sterilisation rate of 75% after 10 years of the 50-year simulation. The island study by Dias, et al. [33] simulated four methods of management on all cats (domestic and feral) on an inhabited island. Current SS methods involved sterilising 21.1% of females and 33.3% of males in the owned population annually. The second method involved sterilising 100% of the owned females and the current 33.3% of the owned males. The third method involved removing (TR) cats of the unowned population annually, so as to reduce it to the same size as the population simulated using current SS methods. The final simulation used the second and third methods concurrently. The current SS method resulted in a 34.3% increase similar to the second method that resulted in a 31.2% increase in the island cat population. The third method required the removal (TR) of 11.7% of the feral population to reduce it to equal the control population (initial population of 50–31.3%). The combined method was the most effective strategy, with a simulated decrease of 2.5% of the initial population. 

## 4. Discussion

A total of 13 articles that reported on cat population management methods were reviewed. Eight articles evaluated in-situ field studies whilst five others used computer simulation models. The articles involving owned cats used surgical sterilisation to manage the cat populations. All articles focusing on free-roaming cats included a form of trap–neuter–return, as either the only method or one of the methods of cat population management, and, therefore, TNR appears to be the most widely accepted, or at least the most widely reported, non-lethal cat population management practice, though it is variable in delivery. Collectively, these TNR studies report varying degrees of success in controlling cat populations, with little to no effect on free-roaming cat populations reported in the short-term TNR programs in urban NYC [21] or rural Quebec [23], whereas the longer term (9–20 years) studies (n = 4) demonstrated significant decreases in free-roaming cat populations in rural and urban areas of the USA and Australia of between 54% and 100% [25,26,27,28]. However, all four long-term TNR programs were supplemented with removal (TR); mainly adoption/rehoming of social cats and the euthanasia of cats that tested positive to FeLV or FIV (feline leukemia and feline immunodeficiency virus respectively). Computer simulations provide greater opportunity to compare different strategies, and investigate the relative contributions of different strategies, when these are used in combination, without the interference of uncontrolled variables. Three articles that simulated TR, as well as TNR, were reviewed. Two of these articles [31,32] reported that TR (even at low intensities) resulted in a higher decrease in cat population than TNR, but did not investigate TR in combination with TNR; whilst the third article [33] only involved TR of the feral cat population of the island (TNR was used on the owned population), precluding a direct comparison. When interpreting the simulation results, it should be noted that these reductions in cat populations were estimated from high-intensity programs conducted frequently (every 6 months) over long periods (10–50 years) and that TR as a sole strategy is not advisable for ethical reasons, nor from a disease epidemiology perspective, as removing cats creates a vacuum that will be filled by immigrant cats [34]. The greater effectiveness of the long-term over the short-term TNR programs reported in the field studies is not surprising. Permanent sterilization does not have an immediate impact on the current cat population, where animals are not removed, but does have a sustainable, longer term impact on the population, as the proportion of the population able to reproduce decreases.

One size does not fit all when it comes to cat management [35]. This likely accounts for at least some of the variation in population changes observed in both the field and simulation results. Simulations accounted for context at different levels, from county-specific to averages across literature and both simulation and field work articles used different frequencies and intensities of TNR for varying durations in different environments. With additional variations in objectives (remove entire population or decrease population size) and population types (owned, urban stray, island populations), it is difficult to suggest ideal targets and specific strategies for TR and TNR. For any context involving owned cats, it would be unrealistic to dictate a TR target, as TR would only be implemented when an owner requests it (i.e., rehoming unwanted cats or kittens or euthanasia where warranted due to injury or sickness). If anything, a target TR rate would be 0% by the end of a program, as it would be expected the population be stable and no unwanted kittens would be born as a result of the TNR program. 

Similar to the remote Indigenous community context, the targeted field TNR program focused on an owned population that had access to subsided veterinary services. However, while this urban UK community had reliable access to veterinary services, remote Indigenous communities have only intermittent, limited access via visiting service providers. Remote communities are difficult to travel to, increasing costs of program delivery, complicating logistics and sometimes dictating methods of program delivery. Transport regulations, for example, often impact on the equipment that can be used in field veterinary hospitals. Remoteness also results in a high turnover of non-local staff (i.e., teachers, health professionals and regional/local government staff) [36,37]. Beyond the significant impacts that this turnover has on the community, the lack of staffing consistency means that veterinary service providers often have to start fresh every trip with a new liaison, make new contacts, train new assistants, etc. This again increases costs, as these processes take time. Importantly, veterinary services focusing on population management rarely address the underlying root causes or indirect impacts of cat ownership. A cat-focused questionnaire of ~50% of households in a remote Indigenous community reported that 94% of the households that had or wanted a cat said the reason was for pest control and in particular to ‘eat the rats’ and/or to ‘keep the snakes away’ [38]. Taking a holistic approach and working with other agencies such as Housing or Pest Control services to mitigate these issues may influence peoples’ motivations for keeping cats and therefore assist in managing cat populations.

Short-term field TNR programs again worked with consistently accessible TNR programs [14,22], except for the Canadian study [24] that had a one-off program. Contrary to the belief that TNR program success is strongly related to the proportion of individuals sterilised, this program had a low and temporary impact on the population, despite a mean of 92% sterilised. This unexpected impact could be explained by immigrant cats that were not accounted for between T_0_ and T_12_, which would increase adult numbers and potentially kitten numbers in the population. The short-term success was most likely due to the TNR program occurring during the breeding season, resulting in interrupted gestations (aborted fetuses) and perhaps a higher kitten mortality rate due to the extreme cold temperatures at this time.

The long-term TNR programs were focused on optimising population decreases or removal of stray cat populations; however, in remote Indigenous communities where cats are owned companions, a more appropriate goal may be the stabilisation of cat populations. The simulation that focused on an owned population [30] reported a 0.68% increase of sterilised cats annually for 20 years, resulting in a sterilised rate of 75%. This 0.68% increase was based on the results of a field work TNR (return to their households) campaign and is an achievable goal in remote Indigenous communities with (albeit limited) access to veterinary services. Another simulation reported that undertaking TNR on 40% of the naïve cats in a population also resulted in a sterilisation rate of 75% after 10 years [32]. To the authors’ knowledge, there is no literature available on a suitable target of the proportion of sterilised cats in a population to reach stabilization of the population. From the interpretations of the authors of the papers reviewed here, it seems that reaching a 75% sterilisation rate is considered necessary to achieve stable cat populations, and that TNR rates (intensity and frequency) will need to differ to achieve this depending on the context of each population. For this reason, monitoring populations is very important to be able to measure the effectiveness of management strategies. To this end, AMRRIC advocates that where veterinary services are occurring in remote Indigenous communities, it is vital that population counts/estimates are conducted (ideally annually) to measure change in population and the proportions of sterilised animals. To do this, AMRRIC conduct a door-to-door census, not only to gather accurate population information but also to engage the community in the process. These censuses are also usually conducted prior to a veterinary service and used to gather a list of households requiring services to make the visit more efficient. AMRRIC has developed a custom-designed database and smartphone/tablet application–The AMRRIC App–to enable such data capture in the field. 

In 2008, Robertson [39] suggested that although there have been some local TNR successes, programs need to be adopted on a far greater scale to have a large impact. Over the last decade, literature regarding TNR programs on free-roaming cats has been growing worldwide. Literature has not only focused on the efficacy of TNR but also on human attitudes and beliefs in relation to free-roaming cat control and the behaviour and welfare of cats, suggesting widespread increasing support for TNR as a tool for managing free-roaming cat populations [40]. 

In Australia, the TNR method is currently being debated [10,41,42,43] as to whether it should be permitted. TNR is currently used unofficially in Australia, mainly by individuals that take semi-owned stray cats (that they feed) to be neutered but not taking full ownership of the cats that are still left to their own devices, and to roam [10]. This is considered illegal in many Australian jurisdictions as it can be construed as returning a feral, invasive species back into the environment [10]. However, AMRRIC works with companion animals in Indigenous communities, with the majority being owned in the unique style mentioned above. As these cats are free-roaming, they are sometimes quite difficult to capture. Sometimes it is as easy as one of the owners picking up the cat and giving it to the veterinarian, but sometimes it is necessary to trap the cat, to ensure the safety of the owners, the veterinarian and the cat. Therefore, TNR seems an appropriate population management method even though the cats are owned and “returned” to their owner(s) rather than to the environment per se.

For the specific, unique context of owned, free-roaming cats in remote Indigenous communities, it seems all three methods of control could be used simultaneously. As the cats are owned, subsidised or free SS may assist in increasing the sterilisation rate of the population. However, as mentioned, many of these cats are free-roaming and differ in their degree of socialisation with humans and therefore TNR is required, with the owners’ permission, to capture these cats for surgery. Furthermore, TR may be used for any injured or ill cats, again with owner permission. There are many challenges associated with rehoming cats to outside locations due to remoteness and transport challenges, typically resulting in exorbitant costs; however, it is worthy of further exploration where animals are consensually surrendered, or as an alternative if euthanasia is not accepted by the owner. Simultaneously implementing these three methods of cat population management generally meet the ethos and cultural practices of remote Indigenous communities in Australia. However, thorough discussions with each individual community and their elders and/or traditional owners before and during implementation is a must to create/maintain positive relationships, which is crucial in sustaining long-term programs. Further research is needed in the frequencies and intensities of each method to stabilise a population. 

This review has captured articles that evaluate efficacy in regard to cat population management methods, however there are other factors that must be considered; community engagement, cultural aspects and logistics (field settings, heat etc.) all of which are interconnected. Community engagement and stakeholder participation is needed to understand the community’s motivations for keeping cats, if they want to continue to have cats, and whether any cultural lore or practices exist that could hinder or strengthen a management program. Stakeholder participation can embrace diverse knowledge and values from all individual stakeholders by engaging them in the decision making [44]. In the eight field articles in this review, no community engagement was reported in terms of deciding which population control method to used. All seven articles focusing on unsocialised cat populations reported TNR as a non-lethal, humane method, whilst the article regarding socialised owned cats involved a population of young cats not yet old enough for surgical sterilisation and were targeted at their local veterinary hospitals. 

In remote Indigenous communities, engaging elders, traditional owners and local rangers provides ecological and cultural knowledge to assist in the logistical planning of a management program that is culturally appropriate. For example, the unique style of cat ownership and the logistics of how to capture them as described above. These factors need to be discussed in great detail with community leaders to ensure culturally appropriate cat population management methods are used. Recognising that many factors vary between communities, consultation needs to be undertaken at the beginning of, and throughout, any project in any community. 

Geographical location of these communities also vary; however, most are in close proximity to natural environments with high biodiversity values. Although Indigenous Australians have lived with and sustainably hunted wildlife for centuries [45], the newly introduced rapidly increasing cat populations in these communities [11,12] has the potential to be catastrophic for the native wildlife. While some work has been done to measure this impact, cats (*Felis* spp.) have quite a cryptic lifestyle [46] and therefore can be difficult to monitor and/or manage [47]. In some remote Indigenous communities, humans still hunt traditionally for their food. The scraps from this food are often fed to pets. Cat scat analyses for diet studies would not be conclusive, as it cannot be determined whether the prey species found in their scat were independently hunted by the cats out in the bush, or whether they were hunted by people and fed to the cats at home. Therefore, tracking movement and roaming patterns have been used as a proxy to monitor spatial and temporal movements of predator and prey species. To the authors’ knowledge, the movement of owned cats in remote communities has only been studied once, with a population of ~80 cats. Three different cats were captured on three different cameras along a track immediately adjacent to native bushland. An additional cat fitted with an i-GotU GPS logger was tracked inside another area of bushland adjacent to the community [11]. All four of these cats were in the bushland during the night at the same time that the native wildlife was active in the same area [11] and, therefore, the potential that these cats were hunting is high.

Due to the explicit context of owned, free-roaming domestic cat population control methods and the multiple factors that can impact on such populations as described above, it is difficult to conduct control/treatment studies with repetition, with little to no variables. There is therefore the limitation of causation in a number of the field work articles reviewed. However, when using simulated computer models, it is difficult to include real-life factors in enough detail to suit a particular context, and in-situ field work studies are generally regarded as producing the most reliable results. Most articles reported the change in population numbers making it easy to compare these results; however, differing intervention intensities and frequencies limit accurate comparisons. This is further limited by articles that use multiple methods (e.g., TNR and TR) concurrently and report results as a whole without differentiating between the results of both methods used. Limited research in this area, as well as the relatively low numbers of available articles to review that met the criteria also limits this review. 

With close proximity to natural environments, further research is also needed on the transition of community cats becoming feral cats and vice versa to get a better understanding of the impact community cats are having on local native wildlife. With many of these cat populations being new, community members are often naïve to the high fecundity of cats and are quickly overwhelmed with multiple cats and kittens, especially in communities lacking access to effective veterinary services delivering population management programs [48]. Some community members have been so overwhelmed by the number of cats breeding in their houses that they are abandoning cats in the native bushland [48]. The owners believe that the cats will survive by hunting for their own food and therefore believe that abandoning them in bushland is the best option. Future research into zoonotic disease prevalence in cat populations is also warranted. With population increases, the potential for humans coming in to contact with cats increases and therefore the spread of zoonotic diseases can also rise. 

## 5. Conclusions

This review has systematically reviewed available literature regarding current global cat population management strategies and discussed the literature’s findings in the context of free-roaming cat populations in remote Indigenous communities. All articles focusing on socialised owned cat populations used SS at subsidised costs, and all articles focusing on unsocialised cat populations implemented TNR (and its variants). It is evident that long term trap–neuter–return programs are more effective than short-term or one-off programs, and that supplementing trap–remove into programs where plausible (e.g., by the adoption of social, unwanted cats and/or the euthanasia of cats that test positive to FeLV or FIV) can improve the efficiency of the program. As the cats in these communities are both free-roaming and owned, implementing all three methods simultaneously is recommended. It is difficult to recommend a regimen for the frequency of TNR programs; however, a sustained 75% sterile population is recommended as the overall target. Therefore, subsidised SS of socialised owned cats or TNR of unsocialized cats supplemented with TR when plausible is recommended as a best-practice, humane method of managing cat populations in remote Australian Indigenous communities. It is also recognised that not one plan will fit all and that further research on the micro-level techniques used to deploy TR (trapping tools and techniques) needs to occur. Culturally appropriate community consultation during all processes is vital in achieving a sustainable management program. 

## Figures and Tables

**Table 1:** Descriptions of the eight criteria for inclusion/exclusion of articles to systemically review current global cat population management practices potentially suitable for the remote Indigenous communities in Australia.

Criteria	Description
Criterion 1	Wrong species i.e., Not ‘*Felis catus*’
Criterion 2	Article was not population management orientated
Criterion 3	Article focused on ex-situ populations, i.e., cats in shelters
Criterion 4	Article was about managing populations in general, i.e., estimating populations or discussing (literature or attitude/perception surveys) management methods rather than implementing them
Criterion 5	Research methods used have not been approved or evaluated as a population management strategy in cats
Criterion 6	Research methods used are not accepted by the residents in the communities that AMRRIC service
Criterion 7	Article did not fully describe their methods of research
Criterion 8	Other: main article not in English

**Table 3:** Results from five articles where the impact of cat population management strategies were estimated through computer model simulation.

Ref.	Duration	Frequency	Description	Environment	Method	Intensity	Population Change (%)	Population Sterilised (%)
[29]	5 years	annually	Free-roaming	Urban	Control	Current spay rate	37.02	
	5 years	annually			TNR	50% more cats 2 months to 10 years	8.31	
	5 years	annually			TNR	50% more 2 months to 5 years	9.89	
	5 years	annually			TNR	50% more 4 months to 5 years	16.72^	
	5 years	annually			TNR	50% more 2 to 6months	16.81	
	5 years	annually			TNR	50% more 4 to 6 months	24.53	
	5 years	annually			TNR	50% more 6 months to 5 years	27.76	
	5 years	annually			TNR	75% more 2 months to 10 years	−1.83	
	5 years	annually			TNR	75% more 2 months to 5 years	0.25	
	5 years	annually			TNR	100% more cats 2 months to 10 years	−9.98	
	5 years	annually			TNR	100% more 2 months to 5 years	−7.49	
[30]	20 years	annually	Owned	Urban	SS	Current spay/neuter rate	Not reported	21.89
	20 years	annually			SS	One campaign in first year then return to current	Not reported	22.57
	20 years	annually			SS	Sterilisation rate of campaign applied annually	Not reported	74.86^
	20 years	annually			SS	0%	Not reported	11.03
	20 years	annually			SS	50%	Not reported	78.36
	20 years	annually			SS	100%	Not reported	87.66
[31]	10 years	every 6 months	Free-roaming	Urban	Control	0%	−0.94	
	10 years	every 6 months			TR	25%	−47.26	
	10 years	every 6 months			TR	50%	−86.4	
	10 years	every 6 months			TNR	25%	−22.5	
	10 years	every 6 months			TNR	50%	−48.4	
[32]	50 years	N/A	Free-roaming	Large/Small Urban	Control	No strategy	18–20% a year until carrying capacity	
	50 years	every 6 months	Free-roaming	Large Urban	TR	10%	−12	
	50 years	every 6 months			TR	20%	−34.5	
	50 years	every 6 months			TR	30%	−76.5	
	50 years	every 6 months			TR	40%	−87.5	
	50 years	every 6 months			TR	50%	−91.5	
	50 years	every 6 months			TNR	10%	−7	
	50 years	every 6 months			TNR	20%	−17	
	50 years	every 6 months			TNR	30%	−35.5	
	50 years	every 6 months			TNR	40%	−50	
	50 years	every 6 months			TNR	50%	−57.5	
	50 years	every 6 months	Free-roaming	Small Urban	TR	10%	−9	
	50 years	every 6 months			TR	20%	−26	
	50 years	every 6 months			TR	30%	−63	
	50 years	every 6 months			TR	40%	−80	
	50 years	every 6 months			TR	50%	−86	
	50 years	every 6 months			TNR	10%	−5	
	50 years	every 6 months			TNR	20%	−9	
	50 years	every 6 months			TNR	30%	−16	
	50 years	every 6 months			TNR	40%	−25	
	50 years	every 6 months			TNR	50%	−33	
	50 years	N/A	Free-roaming	Rural	Control	No strategy	5.5% a year until carrying capacity	
	50 years	every 6 months			TR	10%	−88	
	50 years	every 6 months			TR	20%	−100	
	50 years	every 6 months			TR	30%	−100	
	50 years	every 6 months			TR	40%	−100	
	50 years	every 6 months			TR	50%	−100	
	50 years	every 6 months			TNR	10%	−100	
	50 years	every 6 months			TNR	20%	−100	
	50 years	every 6 months			TNR	30%	−100	
	50 years	every 6 months			TNR	40%	−100	
	50 years	every 6 months			TNR	50%	−100	
[33]	50 years	annually	Owned	Island	SS	Current spay (21.1%) and neuter (33.3%) rate	34.3	
	50 years	annually	Owned		SS	100% owned females + current neuter (33.3%) rate	31.2	
	50 years	annually	Feral		TR	Removal to stabilise (11.7%)	0	
	50 years	annually	Owned and Feral		SS + TR	100% owned females + current neuter (33%) rate + Removal to stabilise (11.7%)	−2.5	

^ Indicates the method that the Authors reported to be the most cost-effective.
